# Early migration patterns of calcium phosphate versus hydroxyapatite-coated stem in uncemented total hip arthroplasty: a prospective randomized clinical trial using radiostereometric analysis

**DOI:** 10.1186/s42836-025-00324-z

**Published:** 2025-08-04

**Authors:** Emelie S. Kristoffersson, Daniel Wästerlund, Anette Nyberg, Sead Crnalic, Kjell G. Nilsson, Volker Otten

**Affiliations:** 1 Department of Diagnostics and Intervention, Orthopedics, Umeå University, 90737 Umeå, Sweden; 2 Department of Nursing, Umeå University, 90737 Umeå, Sweden

**Keywords:** Stem stability, Early migration, Calcium phosphate-coating, Radiostereometric analysis

## Abstract

**Background and purpose:**

The design and coating of uncemented joint implants impact bone ingrowth and thereby the stability of the implant. This prospective randomized clinical trial aimed to compare early migration of two uncemented, similarly shaped femoral stems with either calcium phosphate or hydroxyapatite coating.

**Patients and methods:**

93 patients (102 hips) were randomized to either an intervention calcium phosphate or a conventional hydroxyapatite-coated stem. Migration measurements were performed postoperatively, and at 6 weeks, 3, 12 months, and 2 and 5 years after operation, and analyzed with radiostereometric analysis.

**Results:**

There were no significant differences in migration between the two stems from postoperative to 5-year follow-up. With 6 weeks follow-up as baseline, the calcium phosphate coated stem showed a tendency towards migrating less with a maximum total point motion up to 2 years of 0.84 mm (0.68 to 1.00 95% CI) compared to the hydroxyapatite-coated stem which migrated 1.25 mm (0.99 to 1.52 95% CI) (*P* = 0.010).

**Conclusions:**

Our data show that the calcium phosphate-coated stem is a safe implant in terms of ingrowth stability, and with migration patterns comparable to a hydroxyapatite-coated stem.

## Introduction

Total hip arthroplasty (THA) is a successful treatment for osteoarthritis in the hip, and the incidence of THAs in younger and active patients has increased. [1] Implant longevity depends on fixation quality and wear resistance, with new implant designs and materials constantly being developed to improve function and reduce revision rate. [2] However, not all design modifications have been successful, and in some cases, new implants have shown inferior results. [3, 4].

To reduce the risk of implanting potentially inferior prostheses, stepwise introduction, measuring early migration, and comparing them to well-known implants is mandatory. [5] The initial mechanical stability of the uncemented stem is necessary for secondary bone ingrowth and integration to occur. [6] This emphasizes stem features that ensure immediate stability, including specific designs and coatings. [7]Studies have shown that implant migration within the first two years after surgery is associated with loosening and implant failure. [8–10] Radiostereometric analysis (RSA) of implant fixation is considered the gold standard in migration studies, and due to the high accuracy of the method, it enables a small number of subjects to predict long-term survival. [8, 11–13].

The Link LCU (Waldemar Link GmbH & Co. KG, Hamburg, Germany) femoral component is a new prosthesis with a similar shape to the Corail stem (DePuy Orthopedics Inc., Warsaw, Indiana), which is a commonly used femoral stem with long-term survival of 96.8% at 20 years [14] and 88.4% at 30 years. [15] However, the type and thickness of its coating have been discussed as being comparatively thick (150 μm) and made of pure hydroxyapatite. [16] Compared to Corail, the thickness of the LCU coating is only 15 μm and is made of calcium phosphate applied electrochemically. Together with the large porous basic structure, it is expected to enhance osteoconduction and achieve rapid osseointegration. [17, 18] To our knowledge, there are no earlier studies conducted on early migration comparing calcium phosphate and hydroxyapatite-coated stems.

This randomized clinical trial aimed to assess the migration pattern of the LCU stem to predict its long-term outcome by comparing it with the well-known Corail stem using RSA.

## Materials and methods

### Study setting

This prospective randomized controlled clinical trial was performed between May 2015 and November 2018 at the Department of Orthopedics, Umeå University Hospital, Västerbotten, Sweden. All surgeries were performed at Lycksele Hospital, Sweden, which serves as a center for primary hip and knee arthroplasty surgery in Västerbotten county, producing around 250–300 primary THAs annually. [19].

### Participants and eligibility criteria

Patients referred to our department for osteoarthritis of the hip suited for uncemented hip arthroplasty were consecutively assessed för eligibility. The inclusion criteria were male and non-pregnant female patients aged 18 to 70 years, primary or secondary osteoarthritis of the hip, Dorr type A or B femur, and informed consent to participate in the study. Exclusions criteria were BMI < 18 and > 35 kg/m^2^, inflammatory arthritis (including ongoing oral corticosteriod treatment), diagnosed or treated osteoporosis, Paget’s disease, presence of malignancy, hip dysplasia, previous operation in the same hip, and patients who, assessed by the surgeon, were mentally incapable to comply throughout the study period. A total of 93 patients (102 hips) were included and randomized. Patient characteristics were distributed as described in Table 1.

**Table 1:** Patient demographics

	**LCU (*n* = 47)**	**Corail (*n* = 52)**	
Mean age, yrs (SD)	60.5 (7.3)	60.8 (8.0)	0.849^1^
Sex, female;male	25;22	27;25	0.900^2^
Operated side, left;right	20;27	18;34	0.306^2^
Mean height, cm (SD)	172.0 (0.1)	170.9 (0.1)	0.521^1^
Mean BMI, kg/m^2^ (SD)	27.1 (3.8)	26.6 (4.3)	0.559^1^
Dorr classification			0.166^2^
A	30	26	
B	17	26	
Surgeon			0.396^2^
1	26	28	
2	19	18	
3	2	6	
Median stem position in varus (interquartile range)	1.7 (2.2)	2.0 (3.0)	0.109^1^
Stem size templated vs operated			0.952^2^
≤ ± 1	38	36	
± 2	7	9	
> ± 2	2	2	
Mean time to first RSA, days (SD)	2.8 (0.7)	3.1 (0.7)	0.906^1^

*n*, number of hips. Three patients who were randomized were never operated on, two due to a cancelled operation, and one due to preoperative illness. ^1^ calculated using t-test; ^2^ calculated using chi-square test

### Implants and surgery

**Fig. 1:**
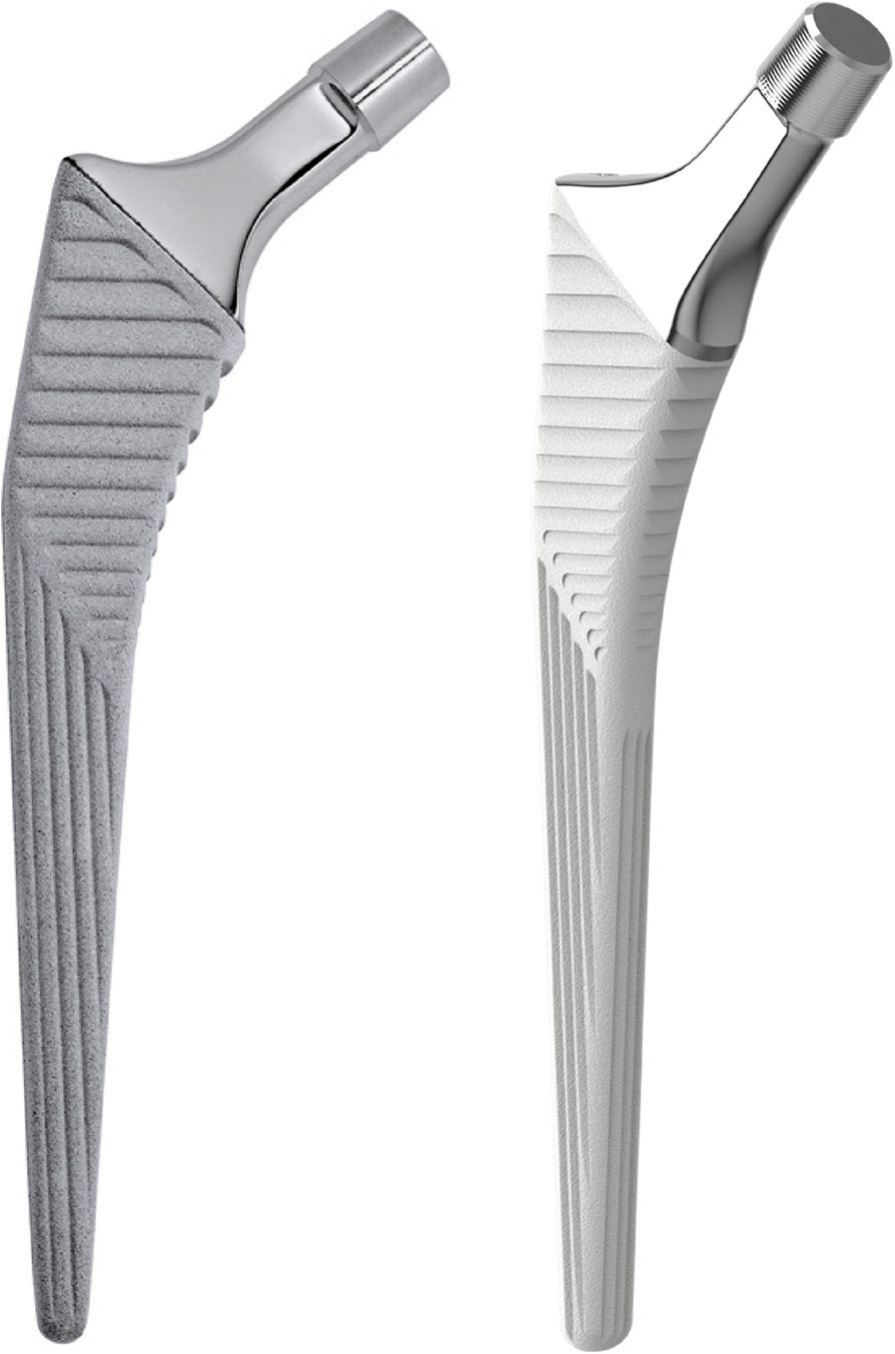
LCU uncemented stem with calcium phosphate coating to the left, Corail uncemented stem with hydroxyapatite coating to the right

The studied femoral components were the uncemented LCU stem (Waldemar Link GmbH & Co. KG, Hamburg, Germany) and Corail stem (DePuy Orthopedics Inc., Warsaw, Indiana). The LCU stem has a similar shape, but with a more angular shoulder, as the straight, tapered, titanium Corail stem (Fig. 1). All LCU stems were collarless. For the Corail stem, all were collarless except for six stems, due to coxa vara hip anatomy, where Corail coxa vara stems (that are only available with a collar) were implanted. The acetabular component was either an uncemented hemispherical, porous-coated, metal-backed cup a Delta TT, Lima Corporate, Udine, Italy) with XLPE liner or Pinnacle (DePuy Orthopedics Inc, Warsaw, Indiana) with Marathon XLPE liner. A 32 mm ceramic head (Biolox delta CeramTec GmbH, Plochingen, Germany) matching the taper of the respective stem was used as standard in all surgeries except for cup sizes less than 48 mm, when a 28 mm ceramic head was used.

All patients were operated on in a lateral position using a posterolateral approach with capsular repair through trans-osseous sutures. The three surgeons involved have long experience with uncemented THA. Postoperatively, all patients were allowed to weight-bear, and rehabilitation started under the supervision of a physiotherapist.

During surgery, nine tantalum markers (1.0 mm in diameter) were implanted into the pelvic bone surrounding the acetabulum, as well as in the lateral and medial aspects of the proximal femur. Additionally, two markers were affixed to the femoral stem: one polyether ether ketone (PEEK) tower was securely attached to the shoulder of the prosthesis, and one marker was cemented to the distal tip just prior to the implantation of the stem. The center of the femoral head served as the third reference point.

### Randomization and blinding

Randomization was performed using an online randomization plan generator (www.randomization.com), which was then put into concealed envelopes. Randomization was stratified for gender. The randomization was done in six blocks, with four possible combinations and an allocation ratio of 1:1. The envelopes were kept separately from the operating theatre and were opened by a research nurse the day before surgery, and the result was conveyed by telephone. The patients, but not the hospital and research staff, were blinded throughout the study period.

### Migration measurements

Migration of the femoral stem was analyzed using RSA. Double examinations for RSA measurements were performed after weight-bearing within the first week postoperatively, and thereafter at 6 weeks, 3, 12, 24, and 60 months. Patients were positioned supine onto a uniplanar calibration cage (UmRSA; RSA Biomedical AB, Umeå, Sweden) with simultaneous exposure from two x-ray tubes (one stationary and one mobile) angled 40 degrees from each other. Analysis of the implant migration was calculated using UmRSA software (version 6.0, RSA Biomedical, Umeå, Sweden). Migration was calculated separately with the postoperative and 6 weeks follow-up examination as baseline reference. In uncemented stems, we expect an initial migration after weightbearing, followed by stabilization. Therefore, we chose a secondary baseline reference at 6 weeks, estimating that the initial migration would already have occurred by then.

Precision was measured on double examinations using the 1-year follow-up as reference. [11] Absolute mean, standard deviation, and 99% confidence interval (CI) were calculated for the recorded migration between two examinations performed within a 15-min interval for all migration directions.

Segment analysis was used to compare the translations and rotations of the femoral component’s gravity center (defined by the three reference points on the femoral stem) relative to markers in the femoral bone along and around three axes: x-axis (medial–lateral), y-axis (proximal–distal), and z-axis (anterior–posterior).

The primary outcome was migration along the y-axis, commonly referred to as subsidence. Secondary outcomes were rotations and/or translations in any other direction.

Patient-reported outcomes were evaluated using the modified HOOS-12 preoperative and after 6 weeks, 3, 12, 24, and 60 months. HOOS-12 is a shortened version of HOOS scoring covering three categories (pain, function, and quality of life). It yields a summarized score ranging from 0 to 100, where 0 is the worst possible outcome and 100 is the best possible outcome. The minimal clinically important changes (MCIC) for HOOS-12 are 25 points. [20].

### Statistical analysis

Sample size calculation was performed using the power analysis software PASS 13 (NCSS Statistical Software, USA).

For stem fixation, a group sample sizes of 36 achieve over 80% power to detect non-inferiority using a one-sided, two-sample t-test to be able to accept a 10% loss to follow-up after 5 years. The margin of non-inferiority is 0.3 with the true difference between the means assumed to be 0.0, to detect the sensitivity limit of 0.2 mm migration from prior studies. The significance level (alpha) of the test is 0.05, and the data are drawn from populations with standard deviations of 0.5 and 0.5.

Statistical analysis was performed using IBM SPSS Statistics (version 29.0.1.0). Patient descriptives were analyzed with standard descriptive statistics, and binary data were calculated using the chi-square test. The RSA data was analyzed for normal distribution using the Shapiro–Wilk test. Data with parametric distribution were analyzed using an independent-sample t-test and presented in means and 95% confidence interval (CI) (99% CI for precision), whereas variables with non-parametric distribution were analyzed using the Mann–Whitney U-test. Scores from patient-reported outcomes were analyzed using the Wilcoxon signed-rank test. Migration analyses were made with signed values. Statistical significance was considered at *P* < 0.05.

## Results

### Patients

**Fig. 2:**
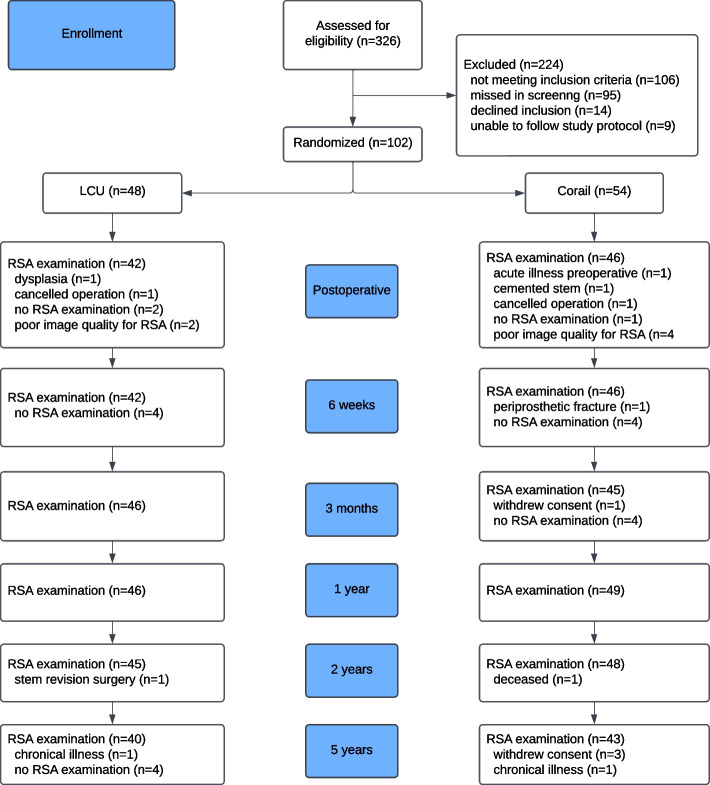
Flowchart according to CONSORT of the study

A total of 93 patients (102 hips) were included and randomized to intervention Link LCU stem or conventional Corail stem and followed throughout the study as described in Fig. 2.

Nine patients with bilateral hip osteoarthritis were included in the study and randomized for both hips. Of these, six patients were assigned to different hip stems, two patients were randomized to receive the LCU stem for both hips, and one patient was randomized to the Corail stem for both hips.

### Precision

Mean condition number, as a measurement of spatial distribution of the tantalum markers, was 40.0 ± 4.4 for the stem and 52.5 ± 43.8 for the femur. The cut-off levels for condition numbers were set at 130. The upper accepted limit for mean error was 0.35.

Precision measurements were performed on 84 double examinations and gave us our measurement bias as presented in Table 2.

**Table 2:** Precision analysis with double examination 1-year follow-up (*n* = 84)

		**Confidence interval 99%**	
	**Mean (SD)**	**Lower**	**Upper**
Translations (mm)			
Mediolateral (x axis)	0.04 (0.00)	0.03	0.05
Proximodistal (y axis)	0.03 (0.00)	0.02	0.04
Anteroposterior (z axis)	0.12 (0.01)	0.09	0.17
Rotations (°)			
Anterior–posterior tilt (x axis)	0.13 (0.16)	0.08	0.17
Anteversion-retroversion (y axis)	0.23 (0.03)	0.17	0.31
Varus-valgus tilt (z axis)	0.05 (0.00)	0.03	0.06

*n,* the number of performed double examinations included in precision measurements

### Primary endpoint

**Fig. 3:**
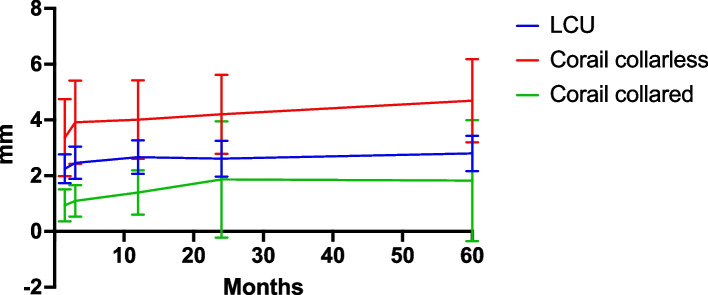
Maximum total point motion (MTPM) with postoperative RSA as baseline (mean ± 95% confidence interval). LCU (*n* = 39), Corail collarless (*n* = 34), Corail collared (*n* = 6)

When analyzing the two stems, we found that the six collared Corail stems had a different migration pattern compared to the collarless Corail stems, as is demonstrated in Fig. 3. The difference in MTPM were greater and statistically significant at 6 weeks follow-up (*P* = 0.031), at 3 months follow-up (*P* = 0.020), and at 1 year follow-up (*P* = 0.044) but not after that. Therefore, we chose to exclude these six stems in analyzing the difference in migration between LCU and Corail.

**Fig. 4:**
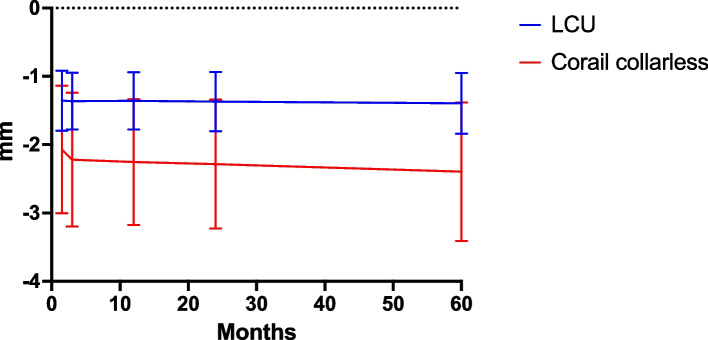
Subsidence (translation along y axis) postoperative to 5 years follow-up presented as mean and 95% CI

**Fig. 5:**
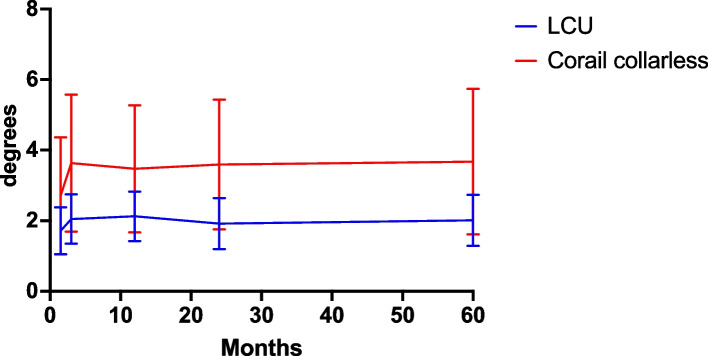
Anteversion-retroversion (rotation around y axis) postoperative to 5 years follow-up presented as mean and 95% CI

The mean migration between postoperative RSA and 2- or 5-year follow-up did not statistically differ in any direction between the two groups, as presented in Table 3 and Figs. 4 and 5. In general, the main migration occurred within the first 6 weeks. From baseline postoperative RSA to follow-up at 6 weeks, there were no statistically significant differences in migration in any direction.

**Table 3:** Migration with postoperative RSA as baseline reference

		**LCU**	**Corail uncollared**
		**Mean (95% CI)**	
Translation (mm)			
Mediolateral (x axis)	6w	− 0.01 (− 0.11 to 0.10)	− 0.03 (− 0.31 to 0.25)
	3 m	0.00 (− 0.09 to 0.10)	− 0.00 (− 0.29 to 0.29)
	12 m	− 0.06 (− 0.16 to 0.04)	− 0.05 (− 0.32 to 0.22)
	24 m	− 0.08 (− 0.18 to 0.02)	− 0.28 (− 0.55 to –0.01)
	60 m	− 0.03 (− 0.14 to 0.08)	− 0.26 (− 0.58 to 0.05)
Proximodistal (y axis)	6w	− 1.36 (− 1.80 to − 0.92)	− 2.07 (− 3.00 to –1.14)
	3 m	− 1.36 (− 1.78 to − 0.95)	− 2.22 (− 3.20 to –1.24)
	12 m	− 1.36 (− 1.78 to − 0.94)	− 2.25 (− 3.17 to –1.33)
	24 m	− 1.37 (− 1.80 to − 0.94)	− 2.28 (− 3.23 to –1.34)
	60 m	− 1.39 (− 1.84 to − 0.95)	− 2.39 (− 3.41 to –1.38)
Anteroposterior (z axis)	6w	− 0.36 (− 0.51 to − 0.21)	− 0.51 (− 0.92 to –0.10)
	3 m	− 0.49 (− 0.71 to − 0.27)	− 0.75 (− 1.21 to –0.29)
	12 m	− 0.37 (− 0.60 to − 0.15)	− 0.59 (− 1.03 to –0.15)
	24 m	− 0.31 (− 0.55 to − 0.07)	− 0.58 (− 1.04 to –0.12)
	60 m	− 0.14 (− 0.36 to 0.07)	− 0.40 (− 0.94 to 0.15)
Rotations (°)			
Anterior–posterior tilt (x axis)	6w	0.01 (− 0.14 to 0.16)	0.05 (− 0.17 to 0.27)
	3 m	− 0.02 (− 0.19 to 0.14)	0.10 (− 0.15 to 0.36)
	12 m	− 0.18 (− 0.38 to 0.02)	− 0.12 (− 0.40 to 0.15)
	24 m	− 0.26 (− 0.47 to − 0.06)	− 0.17 (− 0.42 to 0.08)
	60 m	− 0.57 (− 0.77 to − 0.38)	− 0.55 (− 0.84 to –0.25)
Anteversion-retroversion (y axis)	6w	1.71 (1.05 to 2.38)	2.71 (1.06 to 4.36)
	3 m	2.05 (1.35 to 2.74)	3.63 (1.69 to 5.57)
	12 m	2.13 (1.42 to 2.83)	3.47 (1.67 to 5.27)
	24 m	1.92 (1.19 to 2.64)	3.59 (1.75 to 5.44)
	60 m	2.01 (1.29 to 2.74)	3.68 (1.61 to 5.74)
Varus-valgus tilt (z axis)	6w	− 0.04 (− 0.16 to 0.08)	− 0.17 (− 0.50 to 0.16)
	3 m	− 0.10 (− 0.23 to 0.03)	− 0.17 (− 0.52 to 0.19)
	12 m	0.04 (− 0.09 to 0.18)	− 0.13 (− 0.46 to 0.19)
	24 m	− 0.01 (− 0.15 to 0.13)	0.01 (− 0.31 to 0.33)
	60 m	− 0.09 (− 0.23 to 0.06)	− 0.08 (− 0.41 to 0.25)
Maximum total point motion (MTPM) (mm)	6w	2.25 (1.73 to 2.76)	3.36 (1.98 to 4.74)
	3 m	2.46 (1.88 to 3.04)	3.91 (2.42 to 5.41)
	12 m	2.66 (2.06 to 3.26)	4.01 (2.60 to 5.42)
	24 m	2.61 (1.97 to 3.25)	4.20 (2.78 to 5.62)
	60 m	2.80 (2.16 to 3.43)	4.69 (3.19 to 6.18)

When using 6 weeks of RSA as a baseline, there were statistically significant differences in both anteversion-retroversion rotation (around the y-axis) and maximum total point motion (MTPM) at 12 and 24 months follow-up, where the calcium phosphate-coated stem migrated less, as represented in Table 4.

**Table 4:** Migration with 6 weeks follow-up RSA as baseline reference

		**LCU**	**Corail uncollared**
		Mean (95% CI)	
Mediolateral (x axis)	3 m	0.01 (− 0.02 to 0.03)	0.01 (− 0.02 to 0.04)
	12 m	− 0.01 (− 0.04 to 0.03)	− 0.00 (− 0.03 to 0.03)
	24 m	− 0.01 (− 0.04 to 0.03)	− 0.00 (− 0.04 to 0.03)
	60 m	− 0.02 (− 0.05 to 0.01)	0.01 (− 0.04 to 0.05)
Proximodistal (y axis)	3 m	− 0.02 (− 0.04 to –0.00)	0.01 (− 0.02 to 0.05)
	12 m	− 0.01 (− 0.03 to 0.02)	− 0.02 (− 0.07 to 0.04)
	24 m	− 0.02 (− 0.05 to 0.01)	0.01 (− 0.07 to 0.08)
	60 m	− 0.01 (− 0.05 to 0.03)	0.01 (− 0.06 to 0.08)
Anteroposterior (z axis)	3 m	− 0.02 (− 0.08 to 0.04)	− 0.11 (− 0.20 to –0.02)
	12 m	0.07 (− 0.02 to 0.16)	− 0.04 (− 0.15 to 0.08)
	24 m	0.12 (0.04 to 0.20)	0.08 (− 0.03 to 0.19)
	60 m	0.33 (0.27 to 0.40)	0.23 (0.10 to 0.36)
Anterior–posterior tilt (x axis)	3 m	0.03 (− 0.02 to 0.09)	0.04 (− 0.08 to 0.15)
	12 m	− 0.12 (− 0.22 to − 0.02)	− 0.14 (− 0.32 to 0.04)
	24 m	− 0.19 (− 0.29 to –0.09)	− 0.28 (− 0.50 to –0.06)
	60 m	− 0.40 (− 0.51 to –0.29)	− 0.50 (− 0.68 to –0.33)
Anteversion-retroversion (y axis)	3 m	− 0.03 (− 0.12 to 0.06)	0.21 (0.04 to 0.37)
	12 m	0.04 (− 0.07 to 0.16)	0.25 (0.06 to 0.44)
	24 m	− 0.07 (− 0.18 to 0.04)	0.24 (0.01 to 0.47)
	60 m	− 0.19 (− 0.33 to –0.04)	0.16 (− 0.09 to 0.42)
Varus-valgus tilt (z axis)	3 m	0.01 (− 0.02 to 0.04)	− 0.02 (− 0.08 to 0.04)
	12 m	0.01 (− 0.03 to 0.05)	− 0.05 (− 0.12 to 0.03)
	24 m	0.03 (− 0.02 to 0.08)	0.02 (− 0.07 to 0.10)
	60 m	0.01 (− 0.04 to 0.07)	0.06 (− 0.04 to 0.15)
**Maximum total point motion (MTPM) (mm)**	3 m	0.48 (0.39 to 0.56)	0.73 (0.60 to 0.86)
	12 m	0.83 (0.68 to 0.97)	1.14 (0.92 to 1.35)
	24 m	0.84 (0.68 to 1.00)	1.25 (0.99 to 1.52)
	60 m	1.21 (1.03 to 1.39)	1.43 (1.13 to 1.74)

^*^ *P*-value calculated using the Mann–Whitney U-test due to non-parametric distribution

^a^ *P*-value calculated using Student’s t-test, normal distribution

Eight stems (two LCU, six Corail) migrated more than 0.2 mm in subsidence between 6 weeks and 2 years, and two stems (one LCU, one Corail) subsided more than 0.2 mm from 3 months to 2 years follow-up.

### Revisions

The latest review of patient records screening for adverse events was performed in January 2025. Two cases of revision due to stem loosening were found, one LCU stem and one Corail stem. One patient with diagnosed stem loosening but asymptomatic, who also received a Corail stem. The revised LCU stem showed aseptic loosening after 18 months. When reviewing the postoperative x-ray, the stem was placed in 9° varus, and the stem size implanted was three sizes smaller than templated. The revised Corail stem was positioned in 2° varus, and the implant size was within ± 1 size of the preoperative template. The hip became symptomatic six years after surgery and was revised a few months later.

### Patient-reported outcome

The mean score on preoperative HOOS-12 was 28.8 for LCU and 29.9 for Corail. There was a statistically significant improvement of HOOS-12 in general from preoperative scoring to each follow-up point (*P* < 0.001). When comparing the two groups, we found a statistically significant better scoring in the group receiving the LCU stem at one-year follow-up (*P* = 0.011), where the mean score for LCU was 93.3 and for Corail 87.1, but it was not clinically significant (MCIC < 25).

## Discussion

We found no statistically significant differences in migration patterns between the two studied hip stems postoperatively to two years, which is the recommended outcome period according to the RSA guidelines. [13].

Our results showed a higher degree of migration compared to other migration studies of uncemented stems, especially the degree of subsidence. Campbell et al. conducted an early migration study on 18 Corail hydroxyapatite-coated stems and found a mean subsidence postoperatively to 2 years at 0.58 mm (range –3.71 to 0.23 mm). [21] Direct comparison between studies is challenging because the follow-up baseline may differ, and it is not always clear whether weight-bearing was permitted prior to the initial RSA, which can affect uncemented stems. A defined threshold for end-point migration has yet to be established for uncemented stems. We, as well as others before us, hypothesize that the uncemented stem will migrate after surgery until it osseointegrates into the bone, and that it is after this point that the migration patterns can predict inferior implants. Van der Voort et al. described this as stabilization of migration, suggesting it might be a more accurate focus point for uncemented stems instead of the absolute value. However, further study is needed. [9, 22] This argument is one reason why we added a secondary baseline reference at 6 weeks. Even though the stems in our study migrated more in absolute numbers, the migration patterns after 6 weeks generally showed less than 0.2 mm subsidence. There was one outlier, a collarless Corail stem that subsided 11.2 mm and rotated in anteversion 21.5° between postoperative and 6 weeks follow-up. From 6 weeks to 2 years follow-up, the stem migrated 0.25 mm along the proximodistal axis and rotated 0.43° in anteversion. Exclusion of this outlier did not affect the overall result, as it was included in the migration calculations with further strengthening the results of the calcium phosphate-coated stem. We expect the long-term outcome for the Corail stems in our study to reflect the excellent results seen in registers. Since the LCU stem performed equally well, we also predict a good long-term outcome for this newer implant.

We initially planned for a comparison of collarless stems, but since the Corail coxa vara stem is only available with a collar, the surgeon had no other choice when a coxa vara hip was randomized for Corail than to choose a collared stem. Regarding the migration pattern between collarless and collared Corail stems, the difference was mainly observed in the beginning (Fig. 2) when we expect the ingrowth to happen and when the collar is likely to bring stability. Since there were only six collared stems in our study, there are too few to draw any conclusions, but collars or not have the potential to influence migration.

We are also aware that there are other factors, except our exclusion criteria, that can affect the bone quality and, secondly, the ingrowth stability, such as anticonvulsion and anticoagulation medication, which we have not taken into consideration. [23] The list of medications that could play a role is long and not possible to take into consideration. We rely on the strength of the RCT that these factors will be distributed evenly. The exclusion of these medications would also render our results less clinically relevant due to the risk that our cohort no longer represents the general public in need of a new hip joint.

When isolating rotation around the y-axis using 6 weeks of RSA as baseline, we found some differences favoring the LCU stems up to the 2-year follow-up. The numeric differences were small, and the confidence intervals overlapped, so the clinical value of these findings requires further examination. Potentially, this reduced migration might stem from the thinner coating and enhanced osteoconduction, and that the bone grows into the metal itself and not just the coating. With the thicker HA-coated stems, there are cases of stem loosening with residual ingrowth of the coating, but the metallic stem is loose. A thinner coating might therefore be more favourable and with a longer survival time.

In our cohort, we found two cases of stem revision due to aseptic loosening. We believe the first, early revision was due to unsatisfactory biomechanical factors. The stem was undersized and positioned in varus, two clinically known risks to avoid when implanting hip stems.

Interestingly, when isolating the migration patterns for all loose stems, we saw an initial migration within the first 6 weeks, followed by stabilization and not a continued migration as would be expected with a loose implant. Therefore, we draw the conclusion that migration patterns without provocation should be analyzed on a group level with a sufficient number of patients involved.

The strengths of our study include the fact that we conducted follow-ups over a prolonged period of up to five years, with varying baselines at both the postoperative stage and after 6 weeks. This design allowed for an analysis of migration patterns, encompassing both initial migration and subsequent stabilization. The patient cohort was relatively large, despite some loss to follow-up. Additionally, by comparing similarly shaped uncemented stems, we were able to specifically investigate the effects of different stem coatings.

A weakness in our study was the unequal randomization between the two stem groups. This may have been due to the same cohort being involved in an RCT comparing two acetabular cups, resulting in four possible combinations for randomization. The stratified randomization blocks consisted of 6 blocks instead of 8, which could have statistically led to a more even distribution. Fortunately, the patients lost to follow-up throughout the study made the groups more evenly distributed, and the patient characteristics between the groups were homogenous. Therefore, we estimate the impact of the initially uneven randomization to be low.

## Conclusion

The uncemented calcium phosphate-coated LCU stem is a safe implant in terms of ingrowth stability, and with migration patterns comparable to a hydroxyapatite-coated Corail stem.

## Data Availability

Datasets used and/or analyzed during this study are available from the corresponding author on reasonable request.

## Abbreviations

BMI: Body mass index
CI: Confidence interval
HOOS-12: Hip disability and Osteoarthritis Outcome score-12
MCIC: Minimal clinically important changes
MTPM: Maximum total point motion
PEEK: Polyether ether ketone
RSA: Radiostereometric analysis
THA: Total hip arthroplasty
